# Role of Versican and ADAMTS-1 in Polycystic Ovary Syndrome

**DOI:** 10.4274/jcrpe.3414

**Published:** 2017-03-01

**Authors:** Sibel Özler, Efser Öztaş, Aytekin Tokmak, Merve Ergin, Meryem Kuru Pekcan, Başak Gümüş Güler, Halil İbrahim Yakut, Nafiye Yılmaz

**Affiliations:** 1 Zekai Tahir Burak Women’s Health Training and Research Hospital, Clinic of Perinatology, Ankara, Turkey; 2 Zekai Tahir Burak Women’s Health Training and Research Hospital, Clinic of Obstetrics and Gynecology, Ankara, Turkey; 3 25 Aralık State Hospital, Clinic of Clinical Biochemistry, Gaziantep, Turkey; 4 Liv Hospital, Clinic of Obstetrics and Gynecology, Ankara, Turkey; 5 Zekai Tahir Burak Women’s Health Training and Research Hospital, Clinic of Pediatrics, Ankara, Turkey

**Keywords:** Versican, ADAMTS-1, Polycystic ovary syndrome

## Abstract

**Objective:**

ADAMTS-1 is a matrix metalloproteinase which cleaves versican in the cumulus oocyte complex under the effect of luteinizing hormone surge in the periovulatory period. Altered levels may have a role in the pathogenesis of polycystic ovary syndrome (PCOS). We aimed to determine the serum versican and ADAMTS-1 (a disintegrin and metalloproteinase with thrombospondin motif-1) levels in PCOS patients and compare the results with healthy controls.

**Methods:**

Thirty-eight patients with PCOS and forty healthy controls aged between 15 and 22 years were included in the study. They were sampled according to their basal hormone, serum versican, and ADAMTS-1 levels. Serum versican and ADAMTS-1 levels were measured by enzyme-linked immunosorbent assay. A multivariate logistic regression model was used to identify the independent risk factors of PCOS.

**Results:**

Serum versican levels were significantly decreased in the PCOS group when compared with the controls. The best versican cut-off value for PCOS was calculated to be 33.65 with 76.74% sensitivity and 52.94% specificity. Serum versican levels, homeostasis model assessment of insulin resistance index, a Ferriman-Gallwey score higher than 8, and oligomenorrhea were the strongest predictors of PCOS. Serum versican levels were significantly decreased in PCOS patients. Besides, serum ADAMTS-1 and versican levels were significantly and positively correlated with each other.

**Conclusion:**

Serum versican levels were significantly decreased in patients with PCOS. This suggests a possible role of versican in ovulatory dysfunction and in the pathogenesis of PCOS.

**WHAT IS ALREADY KNOWN ON THIS TOPIC?**

Patients having polycystic ovary syndrome (PCOS), show the symptoms of oligo/anovulation, and hyperandrogenism; and their ultrasonographic examination demonstrates the polycystic view of ovaries. The degradation of cumulus oophorus complex (COC) during ovulation, depends on excretion of various cytokines, prostoglandins, extracellular matrix enzymes, and proteases from the neighboring cells. Versican is one of the proteoglycans forming the COC; and it is degraded by the protease, ADAMTS-1, during the ovulation process.

**WHAT THIS STUDY ADDS?**

Our aim in this study was to investigate if serum versican and ADAMTS-1 levels could be used as a serum marker in anovulation patients. We consider these markers to be used in the treatment strategies of infertile patients having PCOS; with the help of upcoming studies, containing more sample sizes.

## INTRODUCTION

Polycystic ovary syndrome (PCOS) is one of the most frequent endocrinopathies among women of reproductive age, with a prevalence of 6-26%, depending on the diagnostic criteria and ethnicity (1,2). Women with PCOS have increased risk of developing type 2 diabetes. PCOS is also associated with multiple cardiovascular risk factors (3,4). In many women with PCOS, the signs and symptoms start at adolescence (5). Considering the adverse long-term consequences of PCOS, especially when the symptoms begin in the early period of life, early diagnosis and treatment are of great importance for prevention of its progression. It is important to know the underlying factors causing PCOS to provide guidance for early intervention. Although genetic and environmental factors are thought to have a role in the etiopathogenesis of the disease, the exact mechanism is still unclear (5).

PCOS is defined as a syndrome of ovarian dysfunction and its clinical manifestations are mainly attributed to this (4). During both fetal and adult life, continuous remodeling of the ovarian tissue is taking place to maintain normal ovarian function, and this process requires changes also in the extracellular matrix (ECM) (6). Successful ovulation is a complex process consisting of follicular development, ovulation, luteal formation and subsequent regression, and these are all dependent on the cyclical remodeling of ECM (7). Most critical ovulatory mediators were shown to be the ones exerting their effects through the cumulus cell complex surrounding the oocyte (8). Besides, it has been suggested that extensive tissue remodeling of the ovary is mediated by heparan sulfate proteoglycans and matrix metalloproteinases (MMP) (7,9,10).

A disintegrin-like metalloproteinase with thrombospondin motif (ADAMTS) proteases are a distinct group of zinc metalloproteases, comprising 20 members and known to function in the cleavage and degradation of various ECM components (11). Increasing evidence suggests that ADAMTSs have a role in various physiopathological processes such as morphogenesis during embryonic development, follicular development and ovulation, cyclic endometrial remodeling, angiogenesis as well as in development of cancer, of thrombotic and inflammatory conditions (11,12,13,14,15). ADAMTS-1 is a MMP that has been implicated in the inhibition of angiogenesis induced by luteinizing hormone (LH) in the periovulatory follicles of the mouse and rat and is also thought to be a mediator of proteolytic cleavage of the hyaluronan binding proteoglycans, aggrecan, and versican (16,17).

Cumulus oocyte complex (COC) is rich of hyaluronan which is synthesized by cumulus cells, via expression of hyaluronan synthase-2 (18). Versican, a large hyaluronic acid (HA) binding proteoglycan, is expressed by periovulatory granulosa cells and localized within the expanding matrix of COC (18). The secreted active form of ADAMTS-1 has been shown to be localized selectively in COC, and with the effect of LH surge in the periovulatory period, to lead to cleavage of versican (19). Thus, it has been suggested that cleavage of versican in the expanded COC matrix is an important function of ADAMTS-1 in the ovulation process (18,19). Furthermore, previous studies suggested a relationship between versican and atherosclerosis, diabetes, insulin resistance (IR) and endothelial dysfunction, which are well-known long-term consequences of PCOS (3,20,21).

Based on the results of the above-mentioned studies, we hypothesized that altered levels of ADAMTS-1 and versican in the ECM of COC, by causing ovulatory dysfunction, might be causative factors for the clinical manifestations of PCOS. In the present study, we aimed to compare the serum ADAMTS-1 and versican levels in adolescent and young females with PCOS with those of a control group.

## METHODS

Thirty-eight adolescent and young female subjects with PCOS, aged between 15-22 years, were recruited consecutively from the outpatient clinic of Zekai Tahir Burak Women’s Health Training and Research Hospital. The diagnosis of PCOS was based on presence of two of the following three criteria: oligo- or anovulation, clinical and/or biochemical signs of hyperandrogenism, and polycystic ovaries, as proposed by the Rotterdam consensus in 2003 (4). Venous blood from the patients was sampled on the third day of their menstrual cycles. Forty age-matched healthy controls were also recruited.

Exclusion criteria were use of medications known to alter insulin secretion or action and lipoprotein metabolism; hypertension; smoking; family history of cardiovascular diseases; and endocrinopathies including diabetes, Cushing syndrome, androgen-secreting tumors, late-onset 21-hydroxylase deficiency, thyroid dysfunction; current use of oral contraceptives; presence of autoimmune diseases; and hyperprolactinemia. All participants provided a written informed consent and the study protocol was approved by the local ethics committee of our hospital (Approval date/number: 16.10.2014/15).

Clinical examinations were performed and anthropometric measurements were recorded for all participants included in the study. Body mass index (BMI) was calculated by using the formula: weight (kg)/height (m^2^). Waist circumference (WC) was measured as the circumference of the abdomen at its narrowest point between the lower costal (10^th^ rib) border and the top of the iliac crest. Hip circumference was measured at the level of greatest posterior protuberance of the buttocks. Blood samples were obtained by venipuncture after an overnight fasting for at least 12 hours for biochemical evaluation and were processed within 1 hour after withdrawal. The serum samples were stored at -80 °C until the day of analysis.

All analyses were performed with the use of a Beckman Coulter (High Wycombe, United Kingdom) Gen-S automated analyzer. Plasma glucose levels were determined with the glucose hexokinase method. Serum levels of follicle-stimulating hormone (FSH), and LH were determined by immunochemiluminometric assay. Inter-/intra-assay coefficients of variability (CV) for FSH and LH were 2.3%/1.4% and 2.1%/3.1%, respectively. Estradiol, prolactin, dehydroepiandrosterone sulfate (DHEAS), total testosterone (total-T), insulin, and thyroid-stimulating hormone (TSH) were measured using the UniCel DxI 800 radioimmunoassay system (Beckman Coulter, Fullerton, CA, USA). The inter- and intra-assay CVs were 0.1% and 3.2% for estradiol, 1.7% and 3.2% for prolactin, 1.7% and 2.8% for DHEAS, 0.5% and 1.7% for total-T, 8.5% and 2.4% for TSH. Serum levels of 17-hydroxy progesterone (17OH-P) and free testosterone (free-T) were measured by radioimmunoassay (Siemens, Erlangen, Germany). The inter- and intra-assay CVs were 4.6% and 10.7% for 17OH-P and 5.7% and11.4% for free-T, respectively. Homeostasis model assessment of IR (HOMA-IR) (insulin×glycemia in (μmol/L)/ 22.5) was estimated. HOMA-IR >2.5 was considered to indicate the presence of IR (22). The serum levels of total cholesterol, high-density lipoprotein (HDL), low-density lipoprotein (LDL), and triglycerides (TG) were determined with enzymatic colorimetric assays via the use of an AU680 Chemistry System (Beckman Coulter, Fullerton, CA, USA). The lipid accumulation product (LAP) index was calculated using the formula [WC (cm)-58] × [TG concentration (mmol/L)] (23).

Serum ADAMTS-1 concentrations were determined by human ADAMTS-1 enzyme-linked immunosorbent assay (ELISA) (Eastbiopharm Co., Ltd., Hangzhou, China) and the results were expressed as ng/mL. Serum versican concentrations were determined by using human versican ELISA kit (Boster Biological Technology Co., California, USA) and the results were expressed as pg/mL.

## Statistical Analysis

Data analysis was performed by using Statistical Package for the Social Sciences for Windows, version 11.5 (SPSS Inc., Chicago, IL, United States). The data were shown as mean [95% confidence interval (CI)] or number of cases and (percentage), where applicable. The Kolmogorov-Smirnov test was used to determine whether continuous variables were normally distributed or not. Homogeneity of variances was evaluated by the Levene test. Continuous variables were shown as mean ± standard deviation (SD) or median (minimum-maximum), where applicable. Mean differences between case and control groups were compared by student’s t-test. Mann-Whitney U-test was used to compare the median values. Nominal data were analyzed by Pearson’s chi-square test. Whether the mean differences between groups were statistically significant or not, were evaluated by analysis of covariance (ANCOVA). Degrees of association between continuous variables were evaluated by partial correlation analyses. The optimal cut-off points of laboratory parameters discriminating groups were evaluated by receiver operating characteristic (ROC) analyses, calculating area under the curve (AUC) as giving the maximum sum of sensitivity and specificity for the significant test. Multiple logistic regression analyses were applied for calculating odds ratios (OR) and 95% CIs for each clinical condition. Linear regression model was used to evaluate the relation of independent variables as clinical and laboratory parameters in the group having PCOS. A p-value less than 0.05 was considered statistically significant.

## RESULTS

A total of seventy-eight participants (38 adolescent and young females with PCOS and 40 age-matched healthy controls) were enrolled in this case-control study. The baseline clinical, endocrinological, and laboratory characteristics are given in Table 1.

Among the laboratory parameters, fasting plasma glucose, insulin, HOMA-IR, estradiol, and LH levels were significantly higher in PCOS group. LAP index (calculated as 29.86 and 14.17 in PCOS and control groups, respectively) was also significantly higher in PCOS patients (p<0.001). The mean value for WC in PCOS group was 77.23±11.87 cm, which was significantly higher than that of the control group, 72.89±7.78 cm (p=0.041). ADAMTS-1 levels were not significantly lower in PCOS group (p=0.959). The serum versican levels of the PCOS and control groups were detected as 54.69 ng/mL and 95.6 ng/mL, respectively, showing that serum versican levels were significantly decreased in PCOS group when compared with the controls (p=0.009).

**Figure 1:**
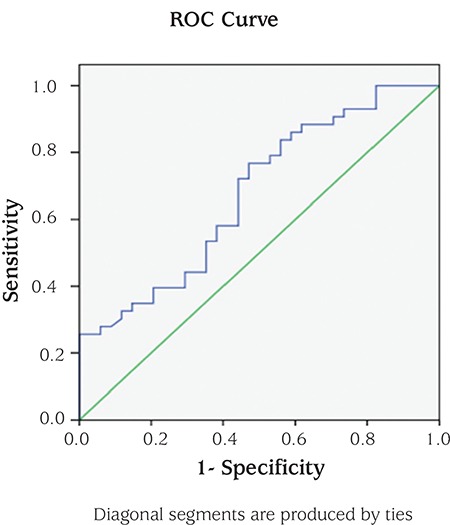
Versican receiver operating characteristic curve. ROC: receiver operating characteristic

**Figure 2:**
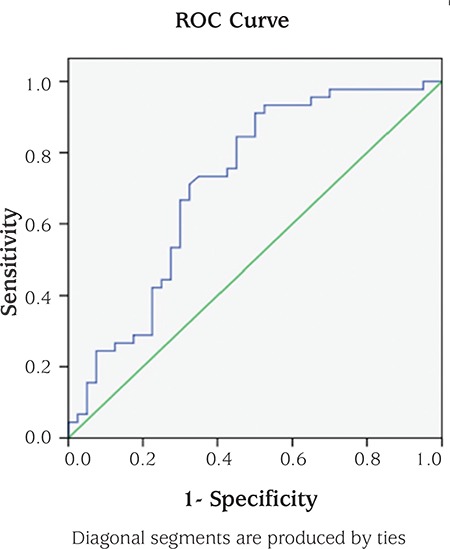
Lipid accumulation product index receiver operating characteristic curve. ROC: receiver operating characteristic

Versican levels were re-evaluated using ROC analysis; cut-off levels were determined and AUC was calculated. According to the ROC analysis performed for the diagnostic performance of serum versican levels for PCOS, the AUC was 0.675 (95% CI: 0.55-0.795; p=0.009) (Figure 1). The best versican cut-off value for PCOS was 33.65 with 76.74% sensitivity, 52.94% specificity, 97.35% positive and 64.29% negative predictive values. According to the ROC analysis performed to assess the differences of LAP index between groups, the AUC was 0.714 (95% CI: 0.601-0.827; p<0.001) (Figure 2).

The best cut-off value of versican for distinguishing PCOS was 16.49 with 63.64% sensitivity, 40.73% specificity, 70.0% positive and 64.44% negative predictive values. All statistically significant parameters according to univariate analysis were further evaluated with multivariate logistic regression analysis (Table 2). Considering the laboratory parameters, versican levels less than 33.65 ng/mL were found to be significantly associated with PCOS (OR: 0.971, 95% CI: 0.949-0.994, p=0.015). Besides, increased levels of LH were also found to be significantly associated with PCOS (OR: 1.247, 95% CI: 1.029-1.513, p=0.024).

We used multiple linear regression analysis with stepwise method to evaluate the predictive effects of independent variables [like serum versican level, HOMA-IR index, Ferriman-Gallwey score (FGS) higher than 8, oligomenorrhea] on PCOS which is a dependent variable. The analysis demonstrated that these parameters were the strongest predictors of PCOS, since they explained 25% of variance in PCOS (Table 3).

In the PCOS group, a statistically significant positive correlation was determined only between ADAMTS-1 and versican levels (r=0.615, p<0.001). No significant correlations were determined between versican levels and HOMA-IR, LAP index, hyperandrogenism, age at menarche, FGS, and oligomenorrhea.

## DISCUSSION

In the present case-control study, significantly decreased levels of serum versican were detected in adolescents and young adults with PCOS when compared with healthy age-matched controls. In addition, a statistically significant positive correlation was determined between serum ADAMTS-1 and versican levels in the PCOS group.

Ovulation requires remodelling of some essential ECM components to permit the release of the COC from the surface of the ovary. This process involves proteolytic events, as well as the proper formation of the expanded COC matrix (24). ADAMTS-1 is shown to be secreted mainly by mural granulosa cells, localized to the ECM of expanded COCs and induced markedly by LH in ovulating follicles (18,25). Besides, versican was also shown to be present in the expanded COC matrix and was induced by LH surge, consistent with the activity and localization of ADAMTS-1 (18,19). Xiao et al (26) investigated the expression of ADAMTS-1 in granulosa cells of the PCOS patients, both by immunocytochemistry and reverse transcription polymerase chain reaction and demonstrated the decreased expression of ADAMTS-1 in PCOS patients when compared with normally ovulating women. On the contrary, we found no statistically significant difference in ADAMTS-1 levels between PCOS and the control groups. Although not significant, ADAMTS-1 levels were lower in the PCOS group and, in the present study, we also demonstrated a significant positive correlation between ADAMTS-1 and versican levels. A possible explanation for this discrepancy may be the limited number of participants of our study. However, Russell et al (18) showed that versican was cleaved in the COCs of the progesterone receptor knocked out mice, even when ADAMTS-1 levels were markedly decreased, a finding which is consistent with the results of the present study.

We observed significantly decreased serum versican levels in PCOS patients when compared with the controls, in our study. Consistent with our result, Richards et al (27) demonstrated that versican was the primary substrate in COCs not only for ADAMTS-1 but also for ADAMTS-4 and 5, and they were all expressed in spatiotemporal patterns suggesting evident and probable overlapping functions with each other. So in the present study, as the versican levels were significantly decreased in the PCOS group, we suggested that it was the primary component of the COCs cleaved during ovulation by the induction of LH surge. However, the insignificantly lower levels of ADAMTS-1 which were observed in our study suggested its important but not the only role in the degradation of ECM components.

LAP index is a cheap and easily available marker of risk for cardiovascular disease. Previous studies have already shown that it has good and reliable diagnostic accuracy for the detection of IR, metabolic syndrome, and risk for cardiovascular disease, even stronger than BMI, WC, and waist-hip ratio (28,29).

We attributed the lack of differences in levels of DHEAS, free-T, total-T, and TG between the groups, to the smallness of our sample. The small number of patients was the main limitation of our study. However, we consider this study important as one of the first in the field and believe that it will encourage further clinical research in this area which include larger samples yielding more significant results.

In conclusion, serum versican levels were found to be significantly decreased in adolescent girls and young women with PCOS. The results of this study support a possible role of versican in ovulatory dysfunction and in the pathogenesis of PCOS.

## Figures and Tables

**Table 1:**
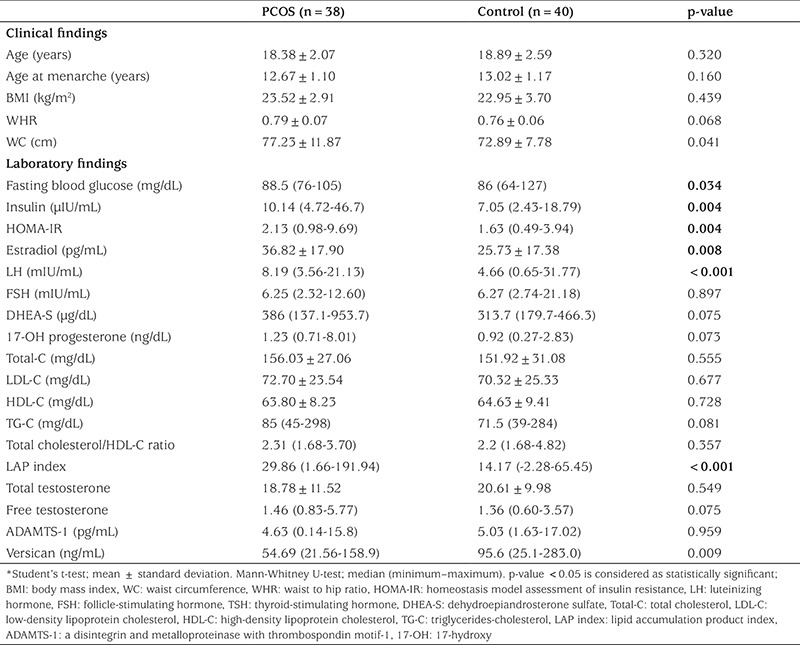
Baseline characteristics, clinical and laboratory parameters of patients with polycystic ovary syndrome and controls

**Table 2:**
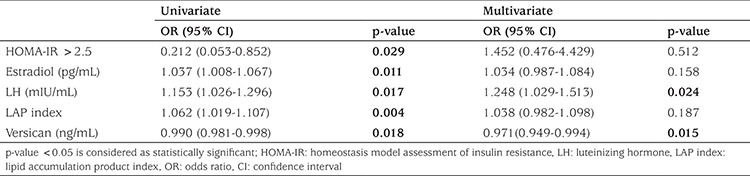
Regression analysis in the prediction of polycystic ovary syndrome

**Table 3:**
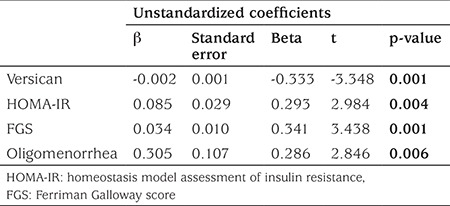
Linear regression model for the assessment of polycystic ovary syndrome risk
